# The Messinian stromatolites of the Sierra del Colmenar (Western Mediterranean): facies characterization and sedimentological interpretation

**DOI:** 10.7717/peerj.5766

**Published:** 2018-10-16

**Authors:** Patricio Guillermo Villafañe, Hugo Corbí, Carlos Cónsole-Gonella, Francisco Javier Ruiz-Sánchez, Jesús Miguel Soria

**Affiliations:** 1 Instituto Superior de Correlación Geológica (INSUGEO), Universidad Nacional de Tucumán-CONICET, Tucumán, Argentina; 2 Department of Earth Sciences and the Environment, Universidad de Alicante, Alicante, Spain; 3 Department of Botany and Geology, Universidad de Valencia, Valencia, Spain

**Keywords:** Stromatolites, Messinian Salinity Crisis, Bajo Segura basin, Neogene, Carbonate sedimentology, Terminal Carbonate Complex, western Mediterranean

## Abstract

A representative outcrop of the Messinian stromatolites belonging to the Terminal Carbonate Complex unit, from the northern sector of the Bajo Segura basin (*Caja de Ahorros del Mediterraneo* section, Sierra del Colmenar, SE Spain) has been studied. Here, we present a detailed analysis of the architecture, external morphology, and internal morphology in order to reconstruct the environmental and palaeoecological conditions for their growth. The stromatolites macrostructure consists of a continuously doming type morphology (build up and sheets areas). These developed close to the coast and acted as a palaeogeographic barrier, reducing physical stress, channeling the erosive effect of water and favoring restricted conditions. This stromatolitic macrostructure exhibits variations in its internal morphology, giving rise to seven subfacies, which are a product of the environmental changes experienced during the growth of the microbial mats. Although broadly suggesting a coastal environment, restricted and shallow during formation, the variation in internal morphology (mesostructure and microstructure) is evidence of minor changes in the physical environment that indicate a progressive shallowing.

## Introduction

The Messinian record of the Bajo Segura basin (western Mediterranean) exhibits well-preserved stromatolites in several areas. These stromatolites were first recorded by Esteban et al. (1978), who reported biohermal stromatolites with hemispherical morphologies of Messinian age in the vicinity of Santa Pola, in Alicante province. Feldmann (1995) and Feldmann & McKenzie (1997) conducted highly descriptive studies in the northern sector of the Bajo Segura basin, defining five different types of stromatolitic structures in Messinian deposits, also in the Santa Pola sector. Later, Soria et al. (2005, 2008b) reported the presence of stromatolites in the northern sector of the Bajo Segura basin, in the Messinian II (MII) synthem, that belongs to the Terminal Carbonate Complex (Calvet, Zamarreno & Valles, 1996), locating them stratigraphically beneath the end-Messinian (e-M) unconformity. Stromatolites have also been reported from the southern sector of the basin, and interpreted as indicators of sudden emersion and subsequent sub-aerial exposure (Corbí, 2010; Corbí et al., 2016).

The studied stromatolites crop out in the Sierra del Colmenar, south of Alicante city, where the *Caja de Ahorros del Mediterraneo* (CAM) sequence is represented (named after a nearby former venue of the banking company CAM; Corbí, 2010). The stromatolites were discovered by a research group from the University of Alicante (Spain) in the late 1990s. Geologically they are included in the northern sector of the Bajo Segura basin, and their stratigraphic sequence belongs, in the terminology of Calvet, Zamarreno & Valles (1996), to the Terminal Carbonate Complex unit (Upper Miocene, Messinian).

The aim of the current study is to understand the relationship between these stromatolites and their sedimentary environment. For this purpose, the stromatolitic facies in this sequence will be defined based on the evaluation of the architecture, external morphology, and variation of internal structure (macrostructure, mesostructure, and microstructure).

### Geological context

**Figure 1:**
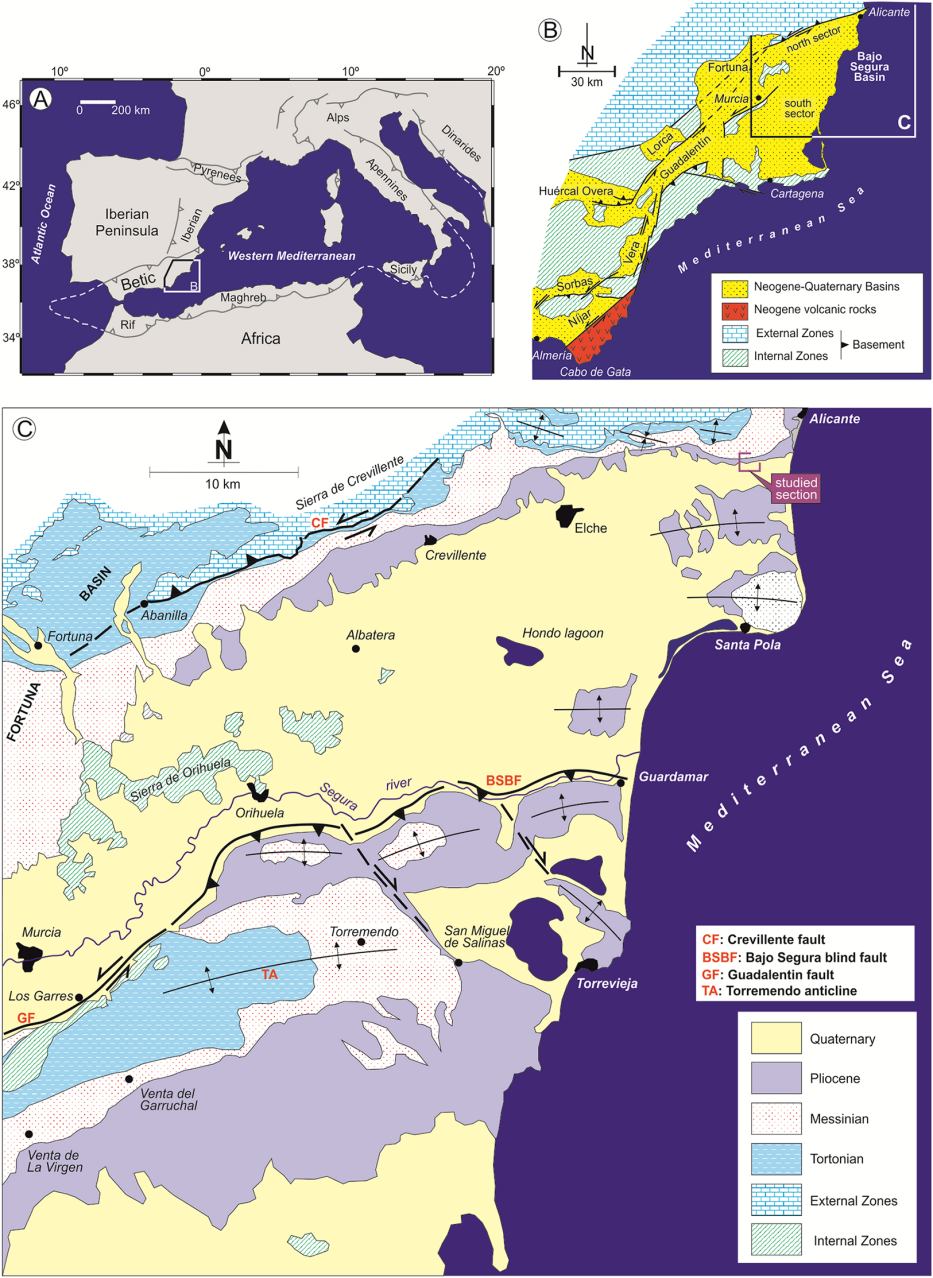
Geological context of the Bajo Segura basin. (A) Location of the Betic Cordillera in the western Mediterranean. (B) Geological map of the Eastern Betic Cordillera showing the location of the Bajo Segura basin. (C) Geological map of the Bajo Segura basin, indicating the position of the studied section.

**Figure 2:**
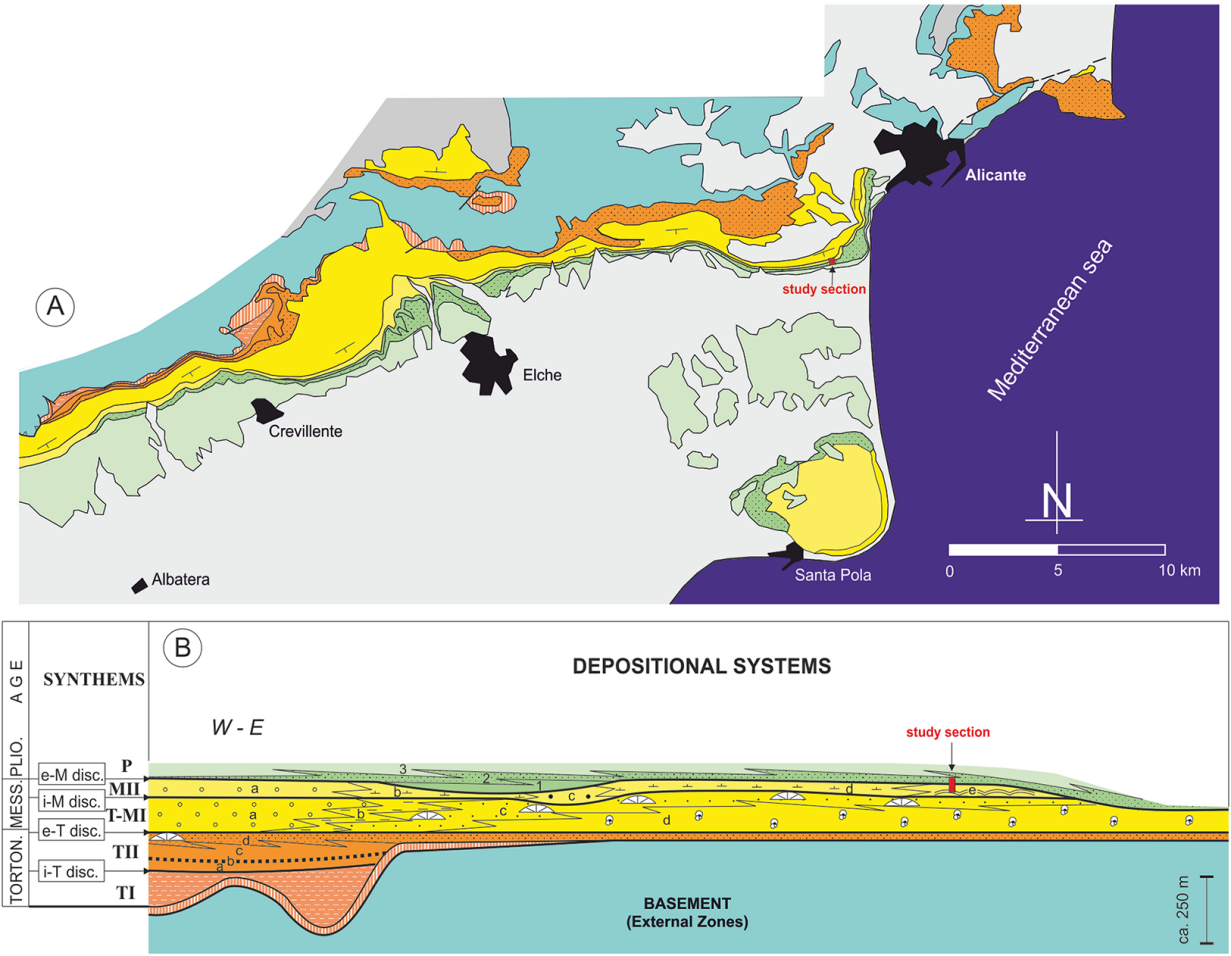
Stratigraphic architecture of the Bajo Segura basin. (A) Geological map of the Bajo Segura basin, indicating the position of the studied section. (B) Synthetic stratigraphic architecture of the northern sector of the Bajo Segura basin, with the location of the study section (for a detailed description Soria et al., 2008a, 2008b; Corbí, 2010; Corbí et al., 2016; Corbí & Soria, 2016). Different colors mark the age of the different units: light blue (Basement, External Zones), orange (Tortonian), yellow (Messinian), green (Pliocene) and gray (quaternary).

The Bajo Segura basin is located at the eastern end of the Betic Cordillera, in southeastern Spain. With a surface area of 3,000 km^2^, it is located in the middle of Alicante Province, except for a small sector in the west of the basin which is included in Murcia Province (Corbí, 2010) (Fig. 1). In this basin, two clearly tectonically and palaeogeographically sectors can be distinguished—North and South—in which the sedimentary record begins in the Tortonian and continues to the Quaternary (Fig. 2). This basin presents one of the most complete Miocene and Pliocene (P) records of the Mediterranean margin, showing an exceptional record of the different sedimentological and palaeoenvironmental phases associated with the Messinian Salinity Crisis (Soria et al., 2008a, 2008b, 2014; Corbí, 2010, 2017; Caracuel et al., 2011; García-García et al., 2011; Soria et al., 2014; Corbí & Soria, 2016; Corbí et al., 2016, 2018). In the northern sector, where our study is focused, the Messinian and Pliocene units present facies associations typical of shallow marine and continental environments; while in the southern sectors, the Messinian and P units record facies associations corresponding to deeper marine environments (Viseras, Soria & Fernandez, 2004).

**Figure 3:**
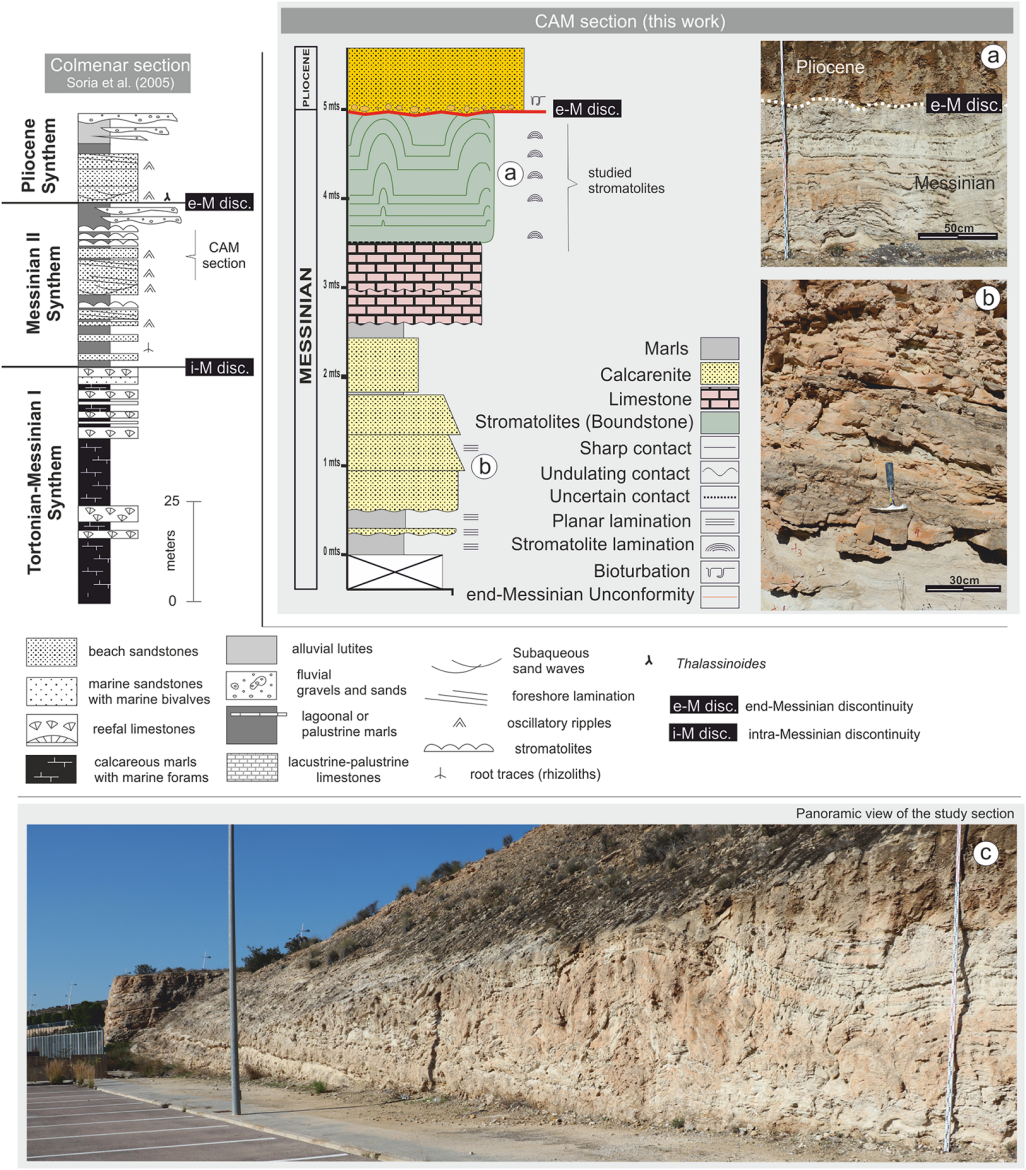
Stratigraphic column for the CAM sequence. (A) Detail of the stromatolitic level and the end-Messinian discontinuity. (B) Alternation of calcarenite strata in the basal sector of the sequence. (C) Panoramic view of the CAM sequence. Photographs taken by Patricio G. Villafañe.

According to the most recent studies (Soria et al., 2008a, 2008b; Corbí et al., 2016; Corbí & Soria, 2016), the marine sedimentary record of the Bajo Segura basin can be divided into five major allostratigraphic units (synthems), termed Tortonian I (TI), Tortonian II (TII), Tortonian-Messinian I (T-MI), MII, and P. These synthems are bounded by basin-wide unconformities: the intra-Tortonian (i-T), end-Tortonian (e-T), intra-Messinian (i-M), and e-M discontinuities (Figs. 2 and 3). The main stratigraphic and sedimentological features of these allostratigraphic units (extensively reported in Corbí, 2010; Corbí & Soria, 2016) are given below. Synthem TI marks the onset of marine sedimentation in the basin. This synthem defines a transgressive-regressive cycle, which consists of a lower deepening-upward sequence (from alluvial-deltaic deposits to pelagic basin marls), and an upper, shallowing-upward sequence (from pelagic basin marls to shallow marine sandstones). Synthem TII (separated from Synthem TI by the i-T discontinuity, expressed by an angular unconformity) is formed by two shallowing-upward, superimposed sequences, both dominated by basin marls topped by shallow marine sandstones. Synthems T-MI and TII are separated by the e-T unconformity, which is a truncation surface. Synthem T-MI comprises a single shallowing-upward sequence, with similar features to the underlying unit. Its upper boundary corresponds to the i-M unconformity. Synthem MII includes both Messinian evaporites and Lago Mare-like sedimentation. The limit with the next synthem (P) is represented by the e-M unconformity, expressed by an erosional surface with palaeovalleys. According to Lancis et al. (2015), the P synthem is represented by two transgressive-regressive marine sequences, which led to the basin’s definitive continentalization.

In the studied area, the most complete stratigraphic section near the study section is the one reported by Soria et al. (2005) and Corbí (2010), named as the “Colmenar” section. Stratigraphically, the stromatolites analyzed in this paper are included in Synthem MII, which is separated from Synthem T-MI by the i-M unconformity and bounded at the top by the e-M unconformity (Soria et al., 2005, 2008a) (Fig. 3). According to these authors, in the northern sector of the basin, Synthem MII comprises five depositional systems: (a) MIIa: red lutites alternating with sandstones and conglomerates, interpreted as deposits of distal alluvial fans with a well-drained floodplain; (b) MIIb: predominantly limestone and marly limestones with gastropod fossils and root bioturbations, intercalated with layers of red clays and dark marls with rodent fossils. These facies associations suggest a lacustrine or palustrine environment without marine influence; (c) MIIc: lutites alternating with marly limestones with rodent fossils and large channels filled with marls and sands, interpreted as a flood zone in a fluvial valley with high-energy channels; (d) MIId: predominantly red and gray marls alternating with massive micritic limestones and channels filled with gravels and sands, interpreted as a coastal lagoon environment interdigitated to the east with beach deposits (MIIe system); and (e) MIIe: recorded only in the Eastern part of the Northern sector of the basin; it presents three different facies associations: calcareous marls with bivalves, stromatolitic limestones forming domes over one m high and oolitic calcarenites with wave ripples. Altogether, the system MIIe represents several coastal sub-environments from the shore face to the backshore and belongs to Terminal Carbonate Complex unit (Calvet, Zamarreno & Valles, 1996).

The top of the Synthem MII coincides with the e-M unconformity, represented by an erosional surface Soria et al. (2008a, 2008b).

## Materials and Methods

The CAM section, located a few kilometers west of the city of Alicante (Fig. 1), was selected for analysis. A detailed stratigraphic column was logged, emphasizing stromatolitic facies(Fig. 3). Material was collected by systematic sampling. Samples were used to prepare thin sections in the Petrology Laboratory at the University of Alicante (Spain) and polished sections in the Industrial Rock Laboratory 1 at the Department of Geology of the University of Valencia (Spain).

Stratigraphic and sedimentological analysis of the sequence followed the methodology proposed by Mercedes-Martin, Salas & Arenas (2014). In addition to facies analysis, field identification of bounding or stratigraphic surfaces were included, which may have regional significance.

The study was performed in two stages. In the first stage, external architecture and morphology were analyzed based on field and laboratory data, including dimensions and spatial distribution of outcrop structures, general external appearance, color, types of contacts and thicknesses. Descriptions were based on the proposals of Clarke & Teichert (1946), Logan (1961), Aitken (1967) and Gebelein (1976); supplemented with descriptive terminology from more contemporary authors such as Davaud, Strasser & Jedoui (1994), Nehza & Woo (2006), Jahnert & Collins (2012), Cooper, Smith & Arnscheidt (2013), Perissinotto et al. (2014) and Suosaari et al. (2016). Added to this were considered the concepts of bioherm and biostrome described by Cumings (1932).

In the second stage, internal morphology was analyzed according to macrostructure, mesostructure, and microstructure. The classification proposed by Logan, Rezak & Ginsburg (1964) was used for the description of macrostructure and the mesostructure (with exception of the cases where lamination is not observed or it is not completely continuous). Hence, the terms used in this work are: (a) LLH-C: close lateral linked hemispheroids; (b) LLH-S: spaced lateral linked hemispheroids; (c) SH-C: vertically stacked hemispheroids with constant basal radius; and (d) SH-V: vertically stacked hemispheroids with variable basal radius.

The macrostructure was distinguished in the field according to textural and lithological characteristics of the stromatolitic facies. Although the main description was based on the classification proposed by Logan, Rezak & Ginsburg (1964), and complemented by the basic structural classification of Organic Reefs and Carbonate Mud Mounds proposed by Riding (2002).

The study of the mesostructure was based on the description of the internal structure of each stromatolitic subfacies. In the laboratory, hand samples and polished sections were used to determine internal structures, lamination types, geometrical variations, textural features, presence of clastic material, etc. This description was based on the classification proposed by Logan, Rezak & Ginsburg (1964) and supplemented with descriptive criteria proposed by Malan (1964), Hoffman (1976), Monty (1976), Schneider (1977), Awramik & Vanyo (1986), Acosta, Garcia Hernandez & Checa (1988), Cohen et al. (1997), Shapiro & Awramik (2000), Suarez-Gonzalez et al. (2014) and Suosaari et al. (2016).

Microstructure was analyzed in the laboratory using thin sections to define parameters involved in stromatolite microstructure: lamination types, stacking, lateral, and vertical continuity of lamination, growth dynamics, hiatuses, etc., and this analysis was carried out taking into account the criteria of the authors listed above.

Stromatolite lamination was analyzed following the concepts proposed by Sarjeant (1975), Scholle (1978), Monty (1976), Reid et al. (2003) and Suarez-Gonzalez et al. (2014), by describing factors such as composition, lateral continuity, thicknesses, geometrical arrangement, etc. Study of erosional structures was based on the criteria of Schneider (1977), Scholle (1978) and Cevallos-Ferriz & Werber (1980).

The description of the carbonates was carried out using the scheme proposed by Folk (1959) and complemented with Kendall & Flood (2011). Porosity was examined according to the classification proposed by Choquette & Pray (1970) and the descriptive concepts of Alonso, Esbert & Ordaz (1987).

## Results

### Description of CAM sequence

Stratigraphically, the CAM sequence records the synthems MII and Pliocene of Soria et al. (2005, 2008a) and Corbí & Soria (2016), ranging from late Messinian to early Pliocene. The section includes stromatolites from the Terminal Carbonate Complex of Calvet, Zamarreno & Valles (1996), which are recorded in Synthem MII of the later authors (Figs. 2 and 3).

Lithologically, the studied sequence is largely composed of alternating tabular strata of calcareous marls, calcarenites, and limestones, dipping approximately 15° to SE (Fig. 3). From the bottom of the profile up to approximately 2.5 m, there are predominantly calcareous sandstone lithologies that alternate with layers of lutite. Above 2.5 m, there are micritic limestone strata. This paper examines the detailed stromatolitic structures one of these strata, between 3.5 and 5.0 m. Above this level there is a layer of calcarenite with massive texture. Above the calcarenite layer there is an erosive surface, with a predominantly subhorizontal—though irregular—morphology, which cuts the stromatolitic domes (Fig. 3). This erosive surface, known as the e-M unconformity, was studied in detail by Soria et al. (2008a) and Corbí (2010). It is of great regional and stratigraphic importance because it separates MII synthem (upper Messinian) from the P synthem (early P). This unconformity, related to the Messinian Salinity Crisis record, was generated by subaerial exposure at the margins of the basin (Soria et al., 2005, 2008a).

Above the e-M unconformity, the sequence starts with conglomerates and a sandy matrix and concludes with shallow marine calcarenites that are organized into massive banks (Fig. 3). The conglomerate (lag deposit according to Caracuel et al., 2011) is composed of ferruginized and intensely colonized clasts, which locally include large pectinids and ostreids (free-lying aggregate of oyster balls, *sensu* Taylor & Wilson (2003)), in some cases colonized by endobionts. According to Caracuel et al. (2011) colonized clasts include profiles of both endolithic (clionids, monactinellids, and large bivalves such as *Lithophaga*) and epilithic organisms (*Cirripedia*, *Vermetidae*, *Serpulidae*, *Ostreidae*, *Polychaeta*, and calcareous algae). Additionally, the sandy matrix includes a rich assemble of shallow marine benthic foraminifera, as *Ammonia beccarii*, *Biasterigerina planorbis*, *Cibicides refulgens*, *Elphidium* spp., and *Nonion commune* (Corbí, 2010).

### Stromatolitic outcrop description

The stromatolitic structures developed on an irregular substrate of calcareous composition (micritic limestones) and occur as a continuous bed whose width runs between 3.5 and 5.0 m.

The stromatolite level consists of predominantly of concave–convex shapes formed by large domes that are in direct contact with each other in some places, and connected by the smaller-scale domes in others. The great lateral development of the stromatolitic structure would suggest that we are faced with a biostrome. However, this would not be entirely correct since Cumings (1932) indicates that these structures don’t have topographic reliefs in any moment of their development, and the stromatolites of the CAM sequence have topographic reliefs throughout its development.

**Figure 4:**
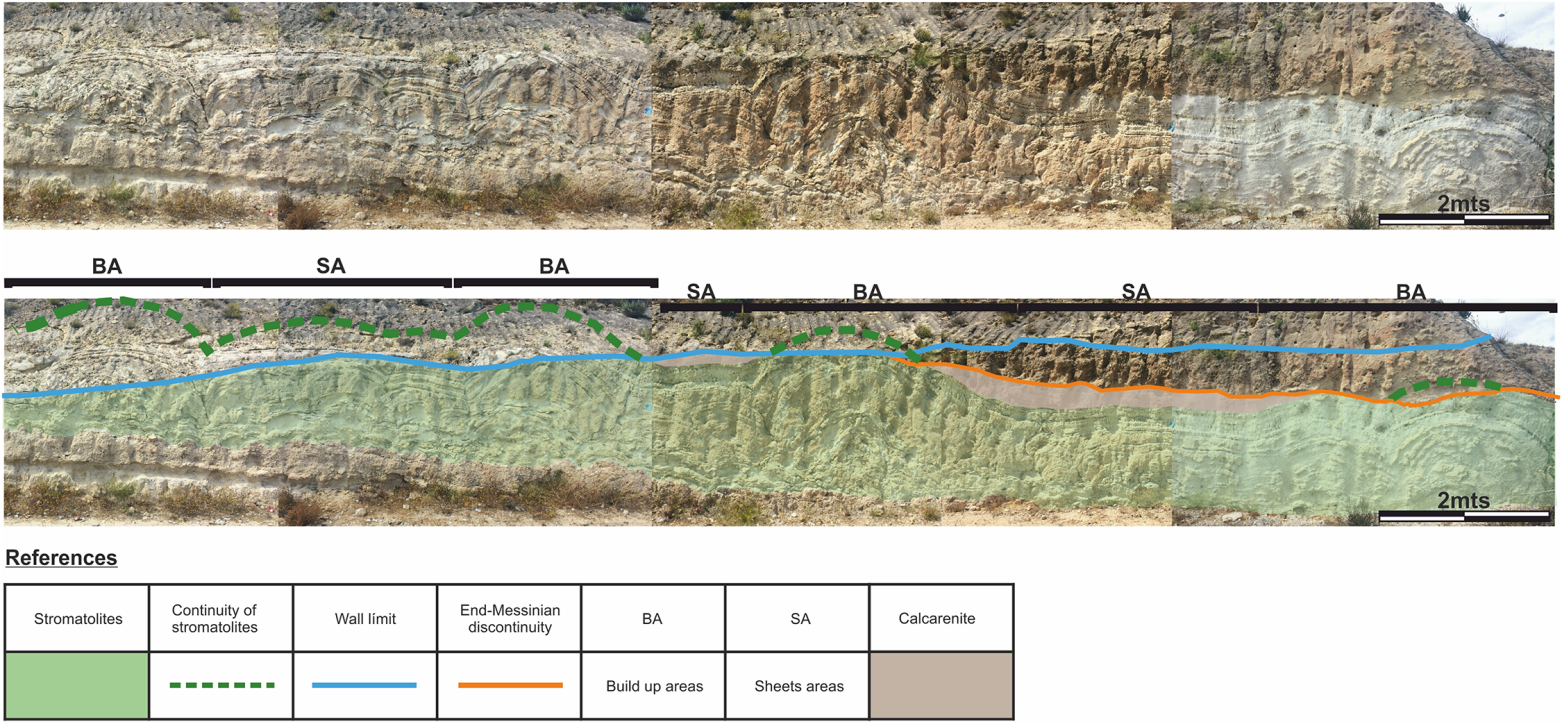
Architecture of the stromatolites. Image worked on a panoramic photograph of the outcrop, which shows the lateral variation in the morphology and architecture of the stromatolites, together with the relationship of these facies with both the substrate on which they developed as well as with the end-Messinian discontinuity. Photographs taken by Patricio G. Villafañe.

To facilitate the description, and to allow a better reference of the sampled areas, the areas of large domes will be called “build up areas” and the areas conformed by the smaller-scale domes that connect the large domes will be called “sheets areas” (Fig. 4).

The “build up areas” have a high synoptic relief, an average height of 2.0 m, a radius of 1.5 m and show a stacking pattern of with a trend of continuous, vertical growth.

The “sheets areas” present minor synoptic relief, an average height of 1.0 m and are 4.0 m long. Although internally they can be composed of both small domes and more mantiform structures, they do not possess the synoptic relief of the “build up areas.”

Both the “build up areas” and the “sheets areas” are stromatolite, which gives to this facies a great horizontal development.

The difference between the synoptic relief of the “build up areas” and “sheets areas” generate depressed areas (lower areas above the “sheets areas”) filled with calcareous sandstones that have massive texture. It should be noted that not all the big domes are fully preserved, as the tops of some of them are cut by the e-M discontinuity, as shown in Fig. 4.

### Internal stromatolite morphology

#### Macrostructure

**Figure 5:**
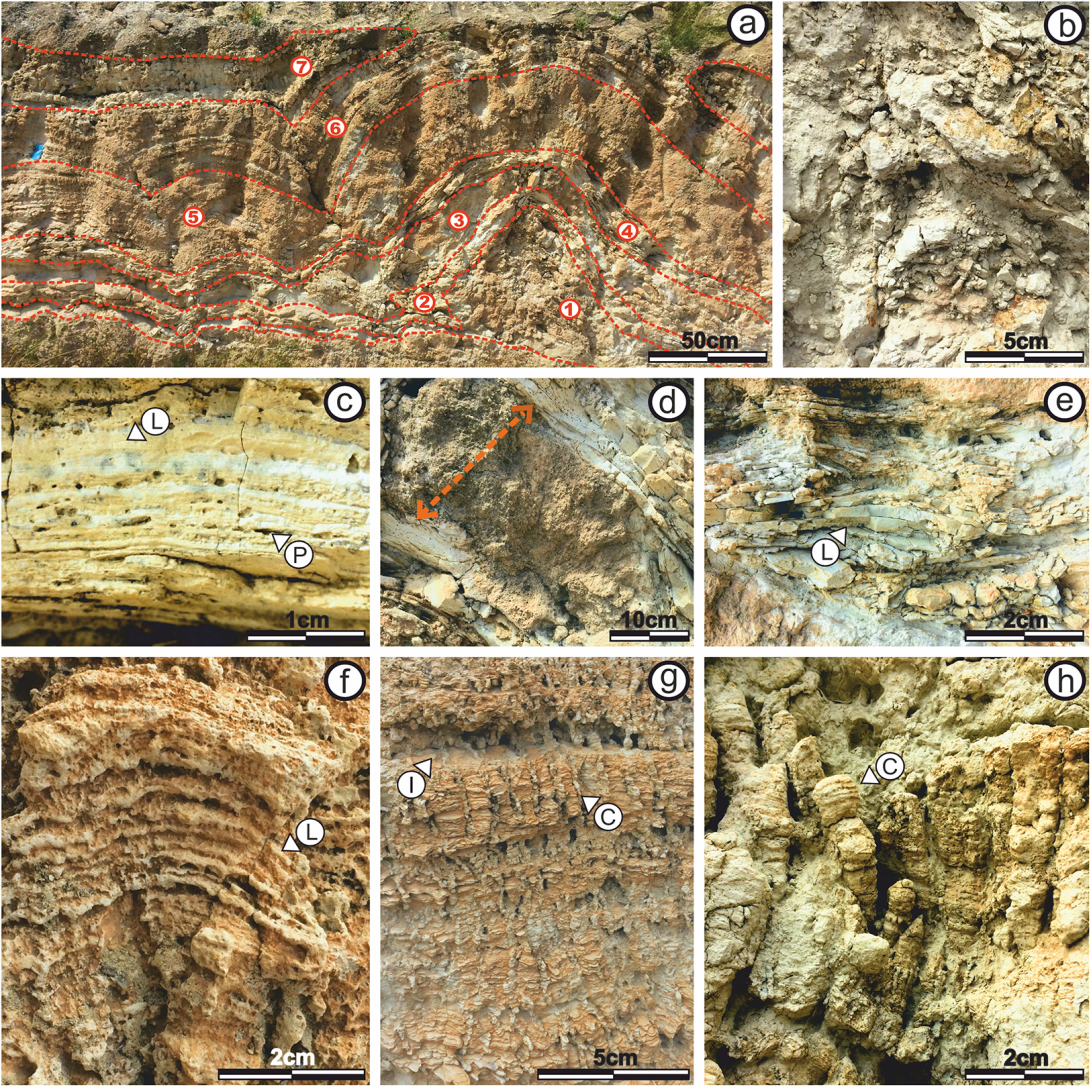
Internal stromatolite morphology: characterization of subfacies. (A) Photograph of the stromatolitic macrostructure in which the seven subfacies that compose it are indicated (numbered from one to seven) and their continuity throughout their horizontal development. It is observed as the continuity of the sufacies make up a macrostructure tipe LLH (B) View of Subfacies 1 in the outcrop. (C) View of Subfacies 2 in the outcrop. Its observed a mesostructure tipe LLH-S. The lamination (L) by which these subfacies are composed and the porosity present. (D) View of Subfacies 3 in the outcrop. The thickness of these subfacies is marked with the dotted line. (E) View of Subfacies 4 in the outcrop. The mesostructure LLH-S shows a lamination (L) with spaced lateral continuity. (F) View in which the Subfacies 5 are shown in the outcrop. The lamination (L) make and close lateral linkage mesostructure (LLH-S). (G) View showing the Subfacies 6 in the outcrop, the columnar structures (C) that compose an SH-C mesostructure and the hiatuses in the lamination (L) growth. (H) View in which the Subfacies 7 are shown in the outcrop and the columnar structures (C) that compose the SH-V mesostructure. Photographs taken by Patricio G. Villafañe.

The stromatolite internal macrostructure morphology shows seven microbialite layers with lateral continuity through the “build up areas” and “sheets areas,” which can be defined as subfacies (Fig. 5A).

The subfacies exhibit lateral linked hemispheroid (LLH) macrostructure (Logan, Rezak & Ginsburg, 1964). In the current study, the zones where “build up areas” are in contact with each other are type LLH-C, while the zones where “build up areas” are connected by “sheets areas” are type LLH-S. For description of their mesostructure and microstructure, the subfacies are numbered from one to seven, following stromatolite growth direction, being (1) the oldest and seven (7) the youngest. In addition, any lateral variation is also mentioned.

#### Characterization of subfacies: mesostructure and microestructure

Subfacies 1: This subfacies is thin (0.15 m) in the “sheets areas,” but reaches up to 0.60 m high in the “build up areas.” The base includes calcareous clasts of up to five mm immersed in micritic matrix (Fig. 5B), in which vuggy porosity (*sensu* Choquette & Pray, 1970) increases upwards in the direction of growth and lamination begins to appear, although it is too diffuse to be classified.

**Figure 6:**
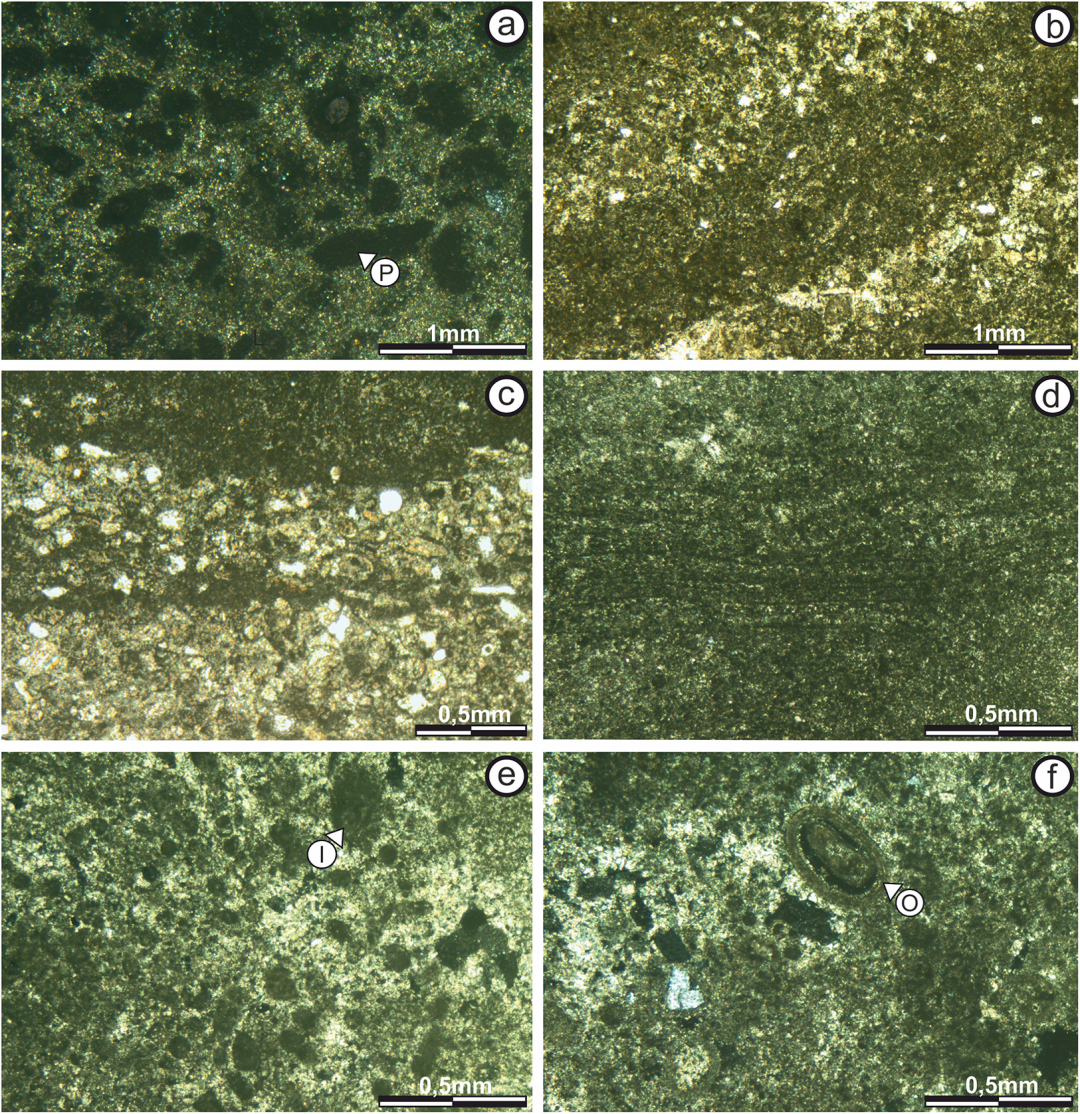
Thin section photographs of stromatolitic Subfacies 1, 2, and 3. (A) Subfacies 1 in the “sheets areas” (image taken with polarized light) showing micritic mud of massive texture with no specific geometry or pattern of arrangement. Empty vuggy pores in dark color (P). (B) In the “build up areas” there are two alternating types of micritic laminae, one with darker dense micritic, and another with lighter micritic and scattered intraclast; giving rise to an alternating simple lamination (image taken with normal transmitted light). The pores are empty and in white color. (C) Subfacies 2 in the “sheets areas” showing alternating micritic and intramicritic laminae (image taken with normal transmitted light). Empty pores in white color. (D) “Build up areas” in Subfacies 2 showing alternation of fine, dark, highly wavy micritic laminae. (E) Presence of intraclasts (I) submerged in the micritic matrix in Subfacies 3 (image taken with polarized light). Empty pores in dark color. (F) Ooid (O) submerged in the micritic matrix in Subfacies 3 (image taken with polarized light). The empty pores are in dark color. Photographs taken by Patricio G. Villafañe.

Microstructure analysis shows that the Subfacies 1, in the “sheets areas,” is composed of micritic mud with massive texture and vuggy porosity (*sensu* Choquette & Pray, 1970) (Fig. 6A). Porosity is 50% of the surface of the section, with diameters ranging from 50 to 400 μm.

In the upper part of the “build up areas” there are two alternating types of micritic laminae, one with darker dense micritic, and another with lighter micritic and scattered intraclast; giving rise to an alternating simple lamination (*sensu* Monty, 1976) (Fig. 6B).

Laminae are 200–500 μm thick, continuous and slightly sinuous, whose boundaries have a diffuse texture (*sensu* Sarjeant, 1975). Porosity in this sector is only 5%, with both vuggy (non-fabric-selective) and fenestral (fabric-selective) types (*sensu* Choquette & Pray, 1970).

Subfacies 2: The mesostructure of Subfacies 2 is characterized by a LLH-S structure (Logan, Rezak & Ginsburg, 1964) which is continuous throughout it (Fig. 5C).

In the “sheets areas” the microstructure shows an alternation between a darker, dense micritic laminae (more than 65%) and lighter-packed, intramicritic laminae (less than 35%), repeating cyclically, and giving rise to an alternating simple lamination (*sensu* Monty, 1976; Fig. 6C).

The thickest laminae (up to 1.5 mm) are those of intramicritic composition, which are composed of micritic intraclasts and some bioclasts (possible foraminifera) immersed in a micritic matrix. The micrite laminae are 50–100 μm thick.

Intraclasts are composed of lithified micritic sediment (100 and 400 μm thick), and surrounded by micritic mud. Bioclasts measure up to 200 μm and are randomly scattered throughout the intramicritic lamination. Porosity is only 35%, and includes vuggy, and fenestral (fabric-selective) types (*sensu* Choquette & Pray, 1970).

In the “build up areas” the alternating simple lamination (*sensu* Monty, 1976) is also observed, it is composed by the alternation of darker dense micritic laminae and lighter micritic laminae with scattered grains (Fig. 6D). Laminae are less than 300 μm thick, laterally continuous, slightly wavy and have boundaries with diffuse texture (*sensu* Sarjeant, 1975), so the contact between successive laminae is transitional.

Porosity is 10%, with non-fabric-selective vuggy porosity and cavities; as well as fabric-selective fenestral type porosity (*sensu* Choquette & Pray, 1970).

Subfacies 3: Subfacies 3 is characterized by the absence of lamination (massively bedded). Its thickness is virtually constant, ranging from 0.15 to 0.25 m (Fig. 5D).

The microstructure is composed by sparse to packed intramicritic texture based on the classification proposed by Folk (1959) and complemented by Kendall & Flood (2011). The intraclasts have micritic composition and sizes over 300 μm. There are sectors with intraclasts either grouped in small pockets up to 600 μm across or scattered randomly throughout the micritic matrix (Fig. 6E). Porosity is not more than 10% of the surface of the section, with vuggy non-fabric-selective porosity being the most common (*sensu* Choquette & Pray, 1970). It is worth noting the presence of sub-rounded, concentric ooids with micritic nuclei, that measure up to 400 μm across, and are scattered randomly within the micritic matrix (Fig. 6F).

Subfacies 4: The mesostructure is characterized by a LLH-S structure (Logan, Rezak & Ginsburg, 1964), which is continuous throughout the entire development of this subfacies (Fig. 5E). The thickness of this subfacies ranges from 0.1 to 0.2 m.

**Figure 7:**
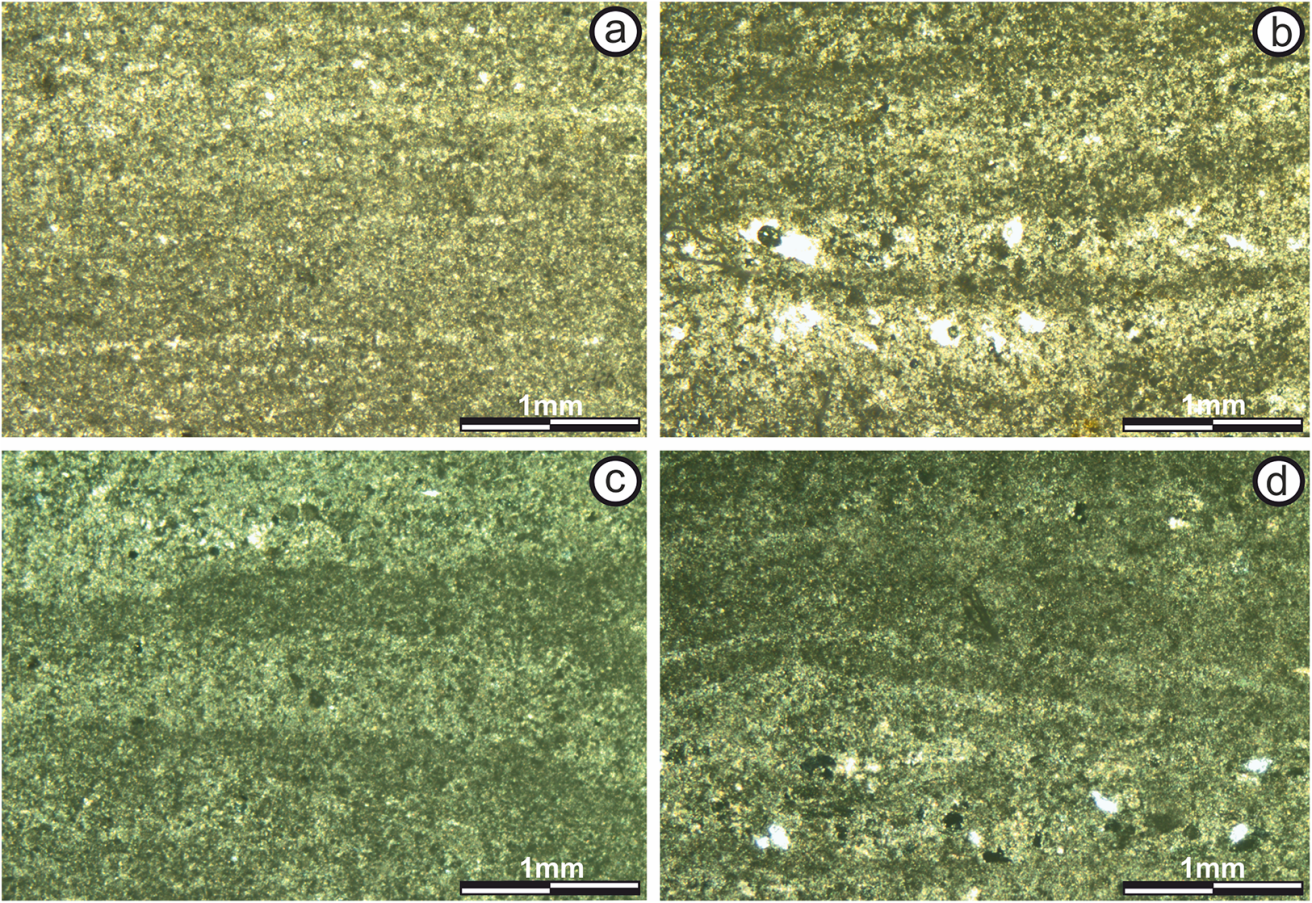
Thin section photographs of stromatolitic Subfacies 4 and 5. (A) Fine micritical film with lateral continuity belonging to the “sheets areas” of Subfacies 4 (image taken with normal transmitted light). (B) “Build up areas” in Subfacies 4 showing alternating lamination of micritic composition (image taken with normal transmitted light). The pores are empty and in white color. (C) Alternation of continuous and sinuous micritic sheets of the “sheets areas” of the Subfacies 5 (image taken with polarized light). The pores are empty and in dark color. (D) “Build up areas” of Subfacies 5 where the alternation of micritic sheets with continuous and sinuous intramicritic sheets is observed (image taken with polarized light). The pores are empty and in dark color. Photographs taken by Patricio G. Villafañe.

In both the “sheets” and “build up” areas, stromatolite microstructure is characterized by the alternation of two types of micritic laminae, darker dense micritic laminae and lighter micritic laminae, giving rise to an alternating simple lamination (*sensu* Monty, 1976; Figs. 7A and 7B). Laminae are up to 200 μm thick, wavy, poor in intraclasts and with diffuse boundaries (*sensu* Sarjeant, 1975), so the contact between successive laminae is transitional (Fig. 7B). Porosity is 2% to 4%, with non-fabric-selective vuggy type morphology (*sensu* Choquette & Pray, 1970).

Subfacies 5: Subfacies 5 is one m thick in the “buil up zone” and up to 0.8 m thick in the “sheets areas,” making this the thickest subfacies. It is characterized by a LLH-C mesostructure (Logan, Rezak & Ginsburg, 1964), which is continuous throughout it (Fig. 5F).

In the interdome area, the microstructure is characterized by an alternating simple lamination (*sensu* Monty, 1976) of darker, dense, micritic laminae, and lighter, micritic laminae. Lamina thickness varies from less than 100 μm to 1 mm; laminae are wavy with diffuse texture boundaries (*sensu* Sarjeant, 1975), and there is transitional contact between successive laminae (Fig. 7C). An average of fields suggests 20% porosity, with pores up to 500 μm and non-fabric-selective vuggy porosity (*sensu* Choquette & Pray, 1970).

The microstructure in the “build up area” is characterized by an alternating simple lamination (*sensu* Monty, 1976) too, but formed by alternating dark, dense, micritic laminae (60%) and packed intramicritic laminae (40%). However, the lamination has no defined order, so it is a simple alternation (*sensu* Monty, 1976) (Fig. 7D). Although micritic laminae are more common, they are thinner (100–300 μm) than intramicritic laminae which can measure up to be one mm thick. Lamination is continuous. Laminae are wavy and have diffuse boundaries (*sensu* Sarjeant, 1975), with transitional contact between successive laminae. Intraclasts present in the intramicrites are smaller than 300 μm, micritic and subrounded.

Porosity accounts for 15% of the total surface of the section and includes both non-fabric-selective vuggy porosity and fabric-selective fenestral and mold porosity (*sensu* Choquette & Pray, 1970).

Subfacies 6: This subfacies has a thickness of 0.25–0.30 m and its mesostructure is defined by a series of columnar structures. These columns are up to 3.5 cm tall and 0.5 cm wide and present constant basal radius (Fig. 5G) giving rise to an SH-C structure (Logan, Rezak & Ginsburg, 1964). The separation between the columns is 0.1–0.2 cm, and the composition of the inter-column space is calcareous sandstones with massive texture.

**Figure 8:**
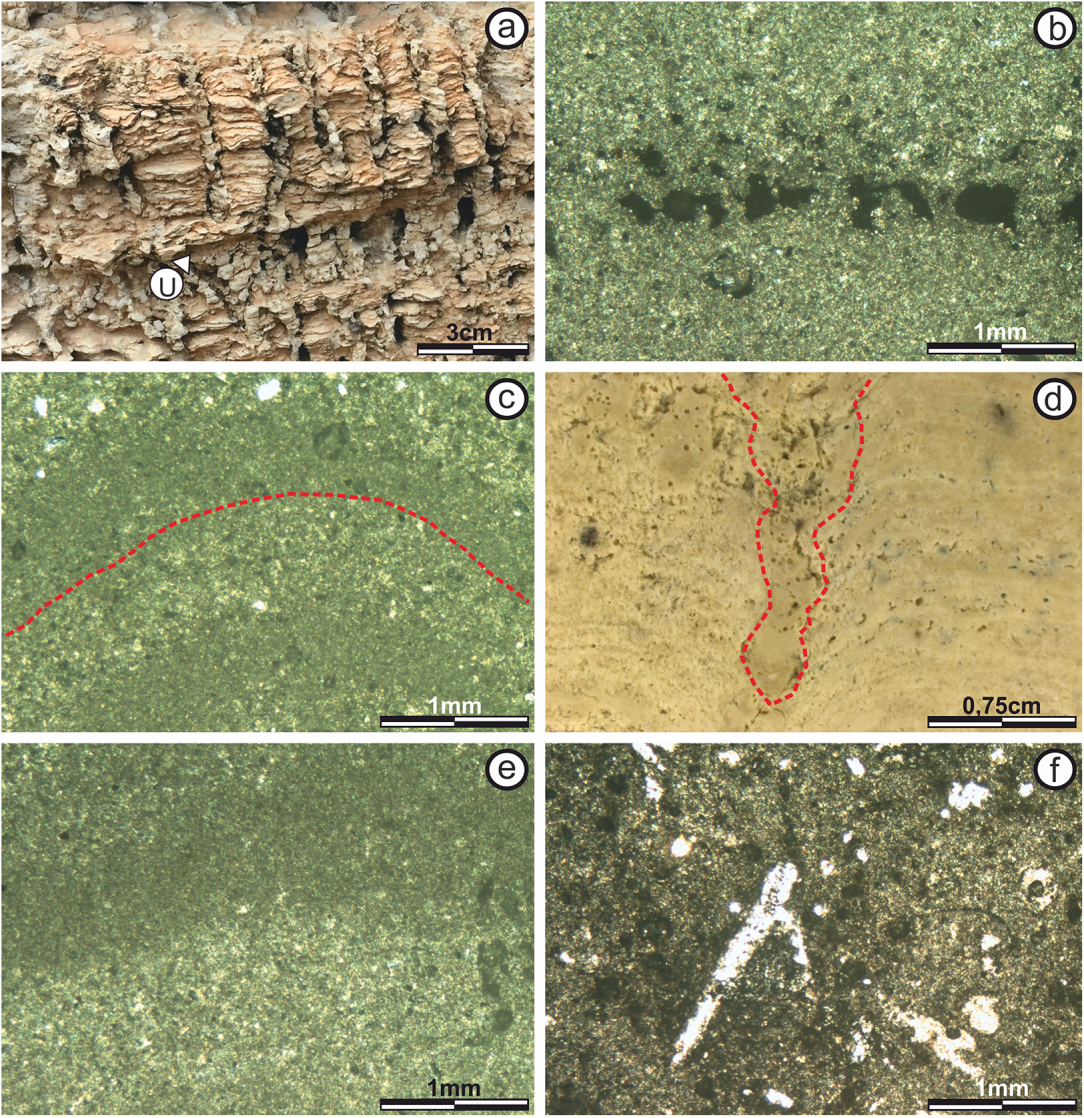
View and thin section photographs of stromatolitic Subfacies 6 and 7. View and thin section photographs of stromatolitic Subfacies 6 and 7. (A) Columnar structures (SH-C) observed in Subfacies 6. (B) Fine micritical film with lateral continuity belonging to the “sheets areas” of Subfacies 6 (image taken with polarized light). The pores are empty and in dark color. (C) Photograph corresponding to Subfacies 6 in the “build up areas” where an alternation is presented of micritic sheets with diffuse edges and concave upward morphology, which are forming the columnar structures (image taken with polarized light). The pores are empty and in dark color. (D) Photograph corresponding to the Subfacies 7, where the space between two columns is marked. (E) Alternating continuous and sinuous micritic sheets of the “sheets areas” of Subfacies 7 (image taken with polarized light). The pores are empty and in dark color. (F) Possible bioperforation observed in Subfacies 7 (image taken with normal transmitted light). Empty pores in white color. Photographs taken by Patricio G. Villafañe.

There are no bifurcations over the vertical development of the columns, and some laminae are observed trying to join two columns. In some cases, there are levels where the vertical growth of the columns is interrupted. This interruption generates horizontal to semi-horizontal surfaces, where calcareous sandstones lacking lamination are recorded. Subsequently the stromatolitic growth is restarted, showing again the development of the columns (Fig. 8A).

The microstructure does not vary between “build up” and “sheets” areas. Laminae are composed of darker, dense micritic laminae and lighter, micritic laminae, with similar patterns (color, thickness and boundaries of laminae), giving rise to an alternating simple lamination (*sensu* Monty, 1976). Lamination is columnar, creating cavities. Most laminae have boundaries with diffuse texture (*sensu* Sarjeant, 1975), so the contact between successive laminae is transitional (Figs. 8A–8C).

Estimated porosity is 20% to 25% with pores of both non-fabric-selective with vuggy and channel porosity (by post-sedimentary tectonic fractures), and fabric-selective with fenestral porosity (*sensu* Choquette & Pray, 1970).

Subfacies 7: Is 0.15–0.20 m thick and is the subfacies where the stromatolite vertical development ends. Its mesostructure is defined by a series of columnar constructions composed of alternating, laterally interrupted calcareous laminae (Fig. 5H). Logan, Rezak & Ginsburg (1964) classify this type of columnar structure as SH type V.

The columnar structures are 0.5–2 cm in diameter, with variable basal radius. There are no bifurcations over the vertical development of the columns, but there are interruption over their vertical growth. This interruption generates horizontal to semi-horizontal surfaces, where calcareous sandstones lacking lamination are deposited. On these surfaces, the growth of the stromatolitic columnar structures begins again.

The columnar structures in this subfacies are more rounded and the separation between the columns is 0.5–1.0 cm (Fig. 8D). The composition of the inter-column space is calcareous sandstones with massive texture.

It should be noted that the “build up areas” in this subfacies is eroded by the late-Messinian unconformity.

The microstructure in this subfacies is characterized by an alternating simple lamination (*sensu* Monty, 1976) of darker, dense, micritic laminae and lighter micritic laminae (Fig. 8E). The laminae are 100 μm to 1 mm thick, wavy and concave upward. Most laminae have boundaries with diffuse texture (*sensu* Sarjeant, 1975), so the contact between successive laminae is transitional. There are lateral bifurcations along some laminae, dividing them in two.

Porosity is 10% with both non-fabric-selective fabric vuggy type morphology and fabric-selective with fenestral porosity (*sensu* Choquette & Pray, 1970). Pore diameters are less than 500 μm (Fig. 8F). Finally, the presence of a possible “bioturbation structure” is recorded in a sector of the thin section, which is developed practically perpendicular to the lamination, towards the interior of the stromatolite. It has a length of 1.6 mm and a width of 0.3 mm. It was the only structure of this type found in these stromatolites.

Using the description of its internal morphology in this terminal section we infer a surface mat of colloform type (Hoffman, 1976; Jahnert & Collins, 2012; Suosaari et al., 2016).

Based on the basic structural classification of Organic Reefs and Carbonate Mud Mounds proposed by Riding (2002), this structure can be classified as “matrix support” for its null skeletal content.

## Discussion

### Main factors in the stromatolic develop

The growth of stromatolites is the result of the interaction between intrinsic and extrinsic factors, which progressively model the final morphology. The extrinsic factors refer to environmental parameters like sedimentary contribution, calcium carbonate saturation, hydrodynamic energy, depth and ecological factors are also considered. While intrinsic factors refer to the microbial mat features (Dupraz, Pattisina & Verrecchia, 2006; Mercedes-Martin, Salas & Arenas, 2014).

#### Sedimentary contribution and calcium carbonate saturation

The sedimentary contribution and the precipitation of carbonates are two very important factors for the stromatolitic growth, since both contribute the “material” for its development (Grotzinger & Knoll, 1999).

The growth of CAM stromatolites was practically carried out independently of the grain supply. The stromatolites grew both with or without significant supply of grain, and when this existed the grains were adhered to the stromatolites surface and colonized by the microbial mucilage (Braga, Martin & Riding, 1995).

The predominance of micritic grain-less laminae during the development of the stromatolite suggest microbially-influenced carbonate in-situ precipitation as the main growth factor (Reid et al., 2000, 2003; Suarez-Gonzalez et al., 2014).

However, the predominance of one process over another will be discussed for each subfacies.

#### Depth

Depth is a very important factor when studying the stromatolitic development, since it can condition it in different ways. On one hand, the depth regulates (along with the morphology of the pre-existing substrate), the accommodation space for stromatolitic growth (Kah et al., 2006). On the other hand, it can directly influence other extrinsic factors such as hydrodynamic energy and light.

The stromatolites in the CAM sequence (based on the internal structure of the Subfacies 7), exhibit a Domical Colloform (*sensu* Jahnert & Collins, 2012) external morphology. Classification of stromatolites based on surface mat type provides limited information of the environmental conditions because they are typically complex structures built by more than one mat type (Hoffman, 1976), but some authors like Hoffman (1976), Jahnert & Collins (2012) and Suosaari et al. (2016) indicate that of colloform structures growth in lower intertidal to subtidal zone at depths of 0.5–2 m.

Although this is valid or not, it would only indicate the depth at the time of development of the seventh subfacies. While authors like Kah et al. (2006) suggest that stromatolites may be sensitive to changes in sea level throughout their development, this is why the depth will be evaluated for each sufacies based on the differences in vertical development, mesostructure, and microstructure.

#### Hydrodynamic energy

Based on the work done by Logan, Rezak & Ginsburg (1964) and Gebelein (1976) we can suggest that both the stromatolitic macrostructure and mesostructure are affected by the hydrodynamic energy of the environment. In addition to this, hydrodynamic energy can influence other factors such as sedimentary contribution and nutrient transport.

In a basin such as Bajo Segura, hydrodynamic energy can be controlled by different factors, including current, waves, climatic events or paleogeographic setting.

In Highborne Cay (Bahamas) Andres & Reid (2006) indicate that the macrostructure of the stromatolites are primarily controlled by hydrodynamic energy with the accommodation space and sedimentation patterns, and in Shark Bay’s (Australia) Suosaari et al. (2016) suggests that stromatolite macrostructure is influenced by currents and associated wave energy. Based on this, these factors can be considered relevant when explaining the macrostructure architecture in the stromatolites of the CAM sequence.

This kind of macrostructure of CAM sequence (LLH) is observed in present-day shallow marine environments near the coastline (Logan, Rezak & Ginsburg, 1964). They also indicate protected locations where wave action is usually mild (Black, 1932; Ginsburg, 1955; Logan, Rezak & Ginsburg, 1964; Suosaari et al., 2016).

Domical shapes would be the stromatolite response to minimize the impact of water acting upon its surface (*sensu* Gebelein, 1976), while the “sheets areas” would serve as large discharge areas (Cevallos-Ferriz & Werber, 1980). Some authors like Feldmann & McKenzie (1997) and Mercedes-Martin, Salas & Arenas (2014), suggest that stromatolitic macrostructures of this size acted as a palaeogeographic barrier, reducing physical stress and erosion, and fostering restricted conditions in response to falling sea level and the increase in the hydrodynamic energy of the environment.

As it was mentioned at the beginning of this section, the mesostructure is also influenced by hydrodynamic energy, and this will be evaluated by the energy changes at the time of the formation of each subfacies.

#### Ecological factors

There is a wide variety of ecological factors that influence and/or control stromatolitic growth. However, given the little evidence about them in the stratigraphic record, we will only focus on the salinity and light.

We can say that salinity is one of the most controversial factors for these stromatolites due to their temporary proximity to the salinity crisis of Messinian. In general, high salinity is a factor that could favor stromatolic growth. This is because a high saline level in the waters increases the environmental stress, reducing both the number of biological populations and species; which under normal conditions compete with stromatolite or can develop activities harmful for this (such as grazing) (Jahnert & Collins, 2012). However, the presence of stromatolites is not necessarily indicative of high saline levels like Riding, Braga & Martin (1991) indicated in Almeria.

Studies on the salinity crisis of Messinian indicate that to know the conditions of salinity is necessary to rely on certain taxa of calcareous nannofossils (Wade & Bown, 2006), or use Sr isotopes (^87^Sr/^86^Sr) to derive more precise palaeosalinity records (Flecker, De Villiers & Ellam, 2002).

In the case of the stromatolites of the CAM sequence, we lack sufficient information to accurately determine the salinity conditions of the water. One possible evidence is its poor fossil content (the only evidence is in the bioclasts of the Subfacies 2, which could be identified as foraminifera). Besides Mercedes-Martin, Salas & Arenas (2014) interprets it in Triassic stromatolites as a result of a progressive increase in environmental salinity, possibly associated with high evaporation rates under restricted arid conditions. While this is applicable for CAM stromatolites, the lack of fossil material could also be caused by other factors such as the lack of nutrients in the water column.

However, based on the work carried out in the Terminal Carbonate Complex unit (Esteban et al., 1978; Calvet, Zamarreno & Valles, 1996), and to the inferences made by Feldmann & McKenzie (1997) on stromatolites of the same age and in the same basin, suggesting that absence of skeletal organisms is an indicator of high salinity, we can estimate that the stromatolites of the CAM sequence could be developed under high salinity conditions.

Other important ecological factors is the light, which in most microbial mat ecosystems is the dominant component that drives the system, except for other essential nutrients needed for biosynthesis (Dupraz, Pattisina & Verrecchia, 2006). Although the degree of luminosity cannot be estimated for the CAM sequence, the kind of stromatolitic macrostructure (LLH) in the sequence is observed in present-day shallow marine environments near the coastline (Logan, Rezak & Ginsburg, 1964), where domes are shallowly submerged and receive good light intensity (Awramik & Vanyo, 1986). In spite of this, the light would probably be controlled by the depth, which would vary for each subfacies.

In addition, other extrinsic factors that cannot be determined with accuracy in the stratigraphic record (nutrient supply, oxygen, temperature, etc.) could have conditioned (to a lesser extent) the final morphology (Dupraz, Pattisina & Verrecchia, 2006; Mercedes-Martin, Salas & Arenas, 2014).

#### Microbial mat

Microbial mats participate actively during stromatolitic growth, both in the “trapping and binding” process like in numerous processes of carbonate precipitation (Riding, 2008).

In turn, the composition of the microbial mat and the way the microbial communities obtain their energy can thus affect global stromatolite morphogenesis, and the lithification process the microestructure (Dupraz, Pattisina & Verrecchia, 2006).

In the studies carried out for this work, it is not possible to observe filaments or any other fossil evidence of the organisms that participated in the growth of stromatolites. However, the abundant, micritic, grain-less laminae suggest a great involvement of microbial activity in their formation (Reid et al., 2000; Dupraz, Pattisina & Verrecchia, 2006).

On the other hand, evidence of microbial activity is proposed, since it possibly originated by retraction parallel to lamination as a result of its desiccation (Alonso, Esbert & Ordaz, 1987), or due to gas bubbles formed during the degradation off organic matter (Mata et al., 2012).

Based on studies carried out on modern stromatolites by Logan, Rezak & Ginsburg (1964), Monty (1976); Awramik & Vanyo (1986), and Andres & Reid (2006), among others, we infer that the stromatolite architecture and morphology observed in the studied stromatolites is a product of variations intrinsic and extrinsic factors during their growth. This interpretation is supported by other studies of the geological record (Riding, Braga & Martin, 1991; Braga, Martin & Riding, 1995; Kah et al., 2006; among others). The relationship between stromatolite morphology and depositional context enables a paleoenvironmental model to be developed.

### Facies interpretation and depositional model

**Figure 9:**
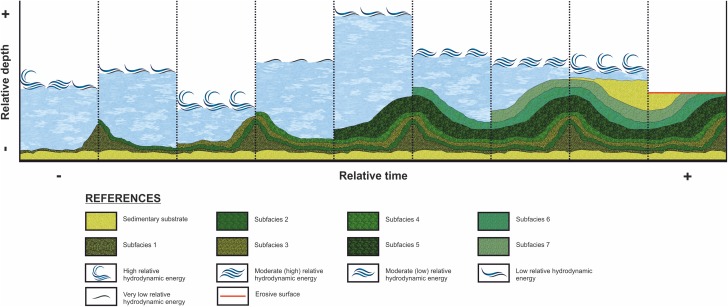
Evolution of the stromatolitic macrostructure over time. While stromatolitic growth is influenced by both extrinsic and intrinsic parameters, according the studied in this paper, the variation of the depth and hydrodynamic energy are considered as the mains factors that influenced the morphology (external and internal) during the growth of the stromatolites of the CAM sequence. This graph show the evolution of the stromatolitic macrostructure over time. In it, the seven subfacies are distinguished which are composing the stromatolite, conditioned by the depth and energy of the water.

In the absence of microbial evidence, the paleoenvironmental reconstruction will focus on determine the physical and chemical parameters that participated in the development of the internal morphology (Fig. 9). The variation in internal morphology over vertical growth provides direct evidence of changes in the environment (Logan, Rezak & Ginsburg, 1964; Dupraz, Pattisina & Verrecchia, 2006; Suosaari et al., 2016).

In the Subfacies 1, the basal sector does not present lamination and consists of massive micrite with calcareous clasts. Reid et al. (2003) interpreted in the Shark Bay stromatolites the unlaminated micrite as a diagenetic microstructure formed by alteration of laminated micrite. However, the presence of clastic material allows to infer the action of hydrodynamic energy (not very high but existing) together with the generation of the micrite.

While upwards in the Subfacies 1, the appearance of micritic laminae and the absence of clastic material indicate a reduction in the relative hydrodynamic energy of the environmental and a primary depositional microstructure formed in a subtidal zone by an unknown mat type (Reid et al., 2000, 2003). Therefore, it may suggest that within these Subfacies 1, the relative hydrodynamic energy decreases from base to ceiling. On top of this subfacies (where the lamination appears for the first time), there are more calmed conditions hydrodynamics conditions allowing micrite precipitation.

On the other hand, stromatolitic lamination for the Subfacies 1 is only evident in the “build up areas.” This indicates that, although they were formed contemporaneously, the hydrodynamic energy was higher in the “sheets areas” than in the “build up areas,” with the “sheets areas” being interpreted as water discharge and runoff channels as observed in similar contexts (Cevallos-Ferriz & Werber, 1980; Mercedes-Martin, Salas & Arenas, 2014). In spite of this, the massive micrite in the “sheets areas” of this subfacies, contemporaneous to the laminae micritic, suggests biological participation with preservation conditions were not the adequate due the greater hydrodynamic energy.

Based on the above, it could be suggested that Subfacies 1 grew in a constantly submerged environment (subtidal) with decresing relative hydrodynamic energy, throughout its development to allow the precipitation of micrite, but even more towards the top where it appears laminated and without clastic material.

For the Subfacies 2, all its mesostructure is of the type LLH-S. Logan, Rezak & Ginsburg (1964) indicates that for this type of structure to develop, a subaqueous environment with relative low hydrodynamic energy is needed, in which stromatolitic mats can develop horizontally without interruption. However, although it is assumed that the conditions are homogeneous, when we observe the microstructure of this subfacies there are indicators of energy variations between the “build up” and “sheets” areas.

The presence of alternating simple lamination (*sensu* Monty, 1976) indicates cyclical variations in environmental control factors throughout the development of this subfacies (Riding, 2008; Decho, 2000; Riding, 2011). But in the “build up areas,” lamination is of micritic composition, indicating that this zone was exposed to lower hydrodynamic energy (Monty, 1976; Reid et al., 2000, 2003; Suarez-Gonzalez et al., 2014) than the “sheets areas,” where micritic and packed intramicritic laminae alternate, indicating time periods with occurrence of temporary increases in the relative hydrodynamic energy of the environment (Scholle, 1978; Kendall & Flood, 2011; Mercedes-Martin, Salas & Arenas, 2014).

This could be explained thinking that through the “sheets areas” the passage of water was channeled, increasing in this area (with respect to the “build up areas”) the hydrodynamic energy, and therefore the sedimentary contribution generating the clastic material in the lamination, during the moments of greater current or waves.

In general line, the Subfacies 2 grew in a shallow subaqueous environment with relative low hydrodynamic energy, where the “sheets areas” acted as channels for the passage of water.

Regarding to Subfacies 3, the absence of lamination can be caused by different factors. One explanation is that the lamination has existed but has been destroyed during the diagenesis (Reid et al., 2003). Other explanation is that during this time intervals the physical, chemical or biological conditions were not appropriate for mat growth (Riding, 2008), or the type of microbial community that was present during that time period did not build laminated structures (Playford et al., 2013; Suosaari et al., 2016).

The sparse to packed intramicritic microstructure indicates waves and current action (Kendall & Flood, 2011). The presence of lithified micrite intraclasts is a result of transportation of autochthonous or para-autochthonous sediment during intervals of greater energy (Acosta, Garcia Hernandez & Checa, 1988; Castro & Ruiz Ortiz, 1991), while the presence of concentric ooids suggests hydrodynamic conditions with high energy in shallow environments (Rohrlich, 1974; Calvet, Zamarreno & Valles, 1996). In addition to this, the lack of evidence of high degree of diagenesis that destroys the preexisting lamination makes the second explanation more plausible.

However, it should be noted that horizontal continuity and constant thickness of this subfacies throughout the entire stromatolite surface suggest biological influence in its generation and can be classified as a thrombolitic mesoscopic texture within a larger laminated microbialite dome (Riding, Braga & Martin, 1991) and similar environmental conditions along a quite homogeneous environmental context.

Based on what was previously described, it could be concluded that the Subfacies 3 were formed in a lower depth environment with respect to the Subfacies 2. This loss in the depth would have favored the action of the waves, increasing the relative hydrodynamic energy of the environment, hindering the growth (or at least the conservation) of the lamination and favoring the presence of intraclasts and concentric ooids.

The Subfacies 4, at the mesostructure level, suggests training conditions similar to Subfacies 2. Since like this one, presents an internal structure of the type LLH-S, which based on the work done by Logan, Rezak & Ginsburg (1964) in Shark Bay, indicates a subaqueous environment with relatively low hydrodynamic energy.

The microstructure in this subfacies also suggests relatively low hydrodynamic energy, since it is entirely composed of micritic grain-less laminae (Reid et al., 2000, 2003; Suarez-Gonzalez et al., 2014); indicating that the accretionary process is controlled by in situ, microbially-influenced carbonate precipitation (Reid et al., 2000).

The decrease in the relative hydrodynamic energy of the environment, with respect to the sub-factories 3, could be explained by an increase in depth, which would decrease the wave action in the system. Another possibility would be an increase in the restriction of the paleogeographic setting, although it is not possible to determine with accuracy whether these factors acted together, or one of them predominated over the other, the decrease in energy in the system is evident (with respect to the other facies), not only due to the predominance of micritic lamination, but also in the absence of clastic material in the “sheets areas.”

The lamination cycle is continuous and uninterrupted, which makes it possible to rule out any sporadic climatic event (Riding, 2008; Decho, 2000; Riding, 2011), which allow us to dismiss extraordinary events such as storms. However, this cyclicity can be related with a seasonal control in fear weather condition.

Based on this, it can be interpreted that the Subfacies 4 was formed in a shallow subtidal environment with relatively low hydrodynamic energy. The decrease in the hydrodynamic energy of this subfacies could be explained by an increase in depth and/or an increase in the restriction of the paleogeographic setting, allowing the generation of micritic lamination throughout its development.

For the Subfacies 5, the LLH-C type mesostructure (Logan, Rezak & Ginsburg, 1964), according to studies in analogous examples, suggests formation in a subtidal environment with relatively low hydrodynamic energy, enabling microbial mats to develop without lateral interruptions (Riding, 2008; Suosaari et al., 2016).

There are no changes in the composition of the laminae, or variations in the quantity or composition of the intraclasts in this subfacies, so it is ruled out that biotic changes or changes in sedimentary supply have been the causes of its substantial vertical growth.

The vertical development of this subfacies could be explained by an increase in water depth, triggering heliotropic growth of microbial mats to ensure better lighting levels (Malan, 1964; Gebelein, 1976; Awramik & Vanyo, 1986; Cohen et al., 1997) and allowing a greater space of accommodation (Kah et al., 2006). It is worth noting that the change from LLH-S (Subfacies 4) to LLH-C type morphology may be explained as a response to the need to increase the specific surface of laminae in order to compensate for the loss of lighting as a result of increasing environmental depth during flooding episodes (Malan, 1964; Gebelein, 1976; Awramik & Vanyo, 1986; Cohen et al., 1997).

The presence of alternating simple lamination (*sensu* Monty, 1976) indicates cyclical variations in environmental factors throughout the development of this subfacies (Riding, 2008; Decho, 2000; Riding, 2011).

In the “sheets areas” of Subfacies 5, the accretionary process is controlled by the in situ, microbially influenced, carbonate precipitation generating micritic grain-less laminae (Reid et al., 2000). On the other hand, in the “build up areas” of this subfacies micritic laminae alternate with packed intramicritic laminae, so that trapping and binding of allochtonous sedimentary material becomes more important in the accretion process. Ginsburg (1955) suggested that the fact that intramicritic laminae are present only in the “build up areas” may be the result of flooding episodes which increased the depth of the environment and generated more active binding of sediment in the parts with greater relief and therefore intramicritic lamination.

It can be concluded that the Subfacies 5 grew up in a subtidal environment with relatively low hydrodynamic energy. Throughout the development of these subfacies, the depth of the environment was increasing, resulting in great vertical growth. Added to this, episodes of periodic inundation would have contributed allochthonous sedimentary material binding in the “build up areas.”

The Subfacies 6 has a mesostructure composed of laminated columns (SH-C internal structure). The presence of columnar structures is probably a response to the energetic conditions of the environment (Dupraz, Pattisina & Verrecchia, 2006), although the influence of biotic factors on them cannot be denied despite that evidence was not found about them (Von Der Borch, Bolton & Warren, 1977).

Dupraz, Pattisina & Verrecchia (2006) indicates that the presence of columnar structures depends on a factor called “attraction distance.” The “attraction distance” is a representation of the attraction force of the build up. Increasing the attraction distance leads to larger protection zones (where no growth is possible) that will produce the formation of wider-spaced columnar or branching morphologies.

The protection zones can be the result of differential erosive effects during discharge and runoff of water along “channels,” truncating the microbial mats in vivo and preventing lateral development of lamination (Schneider, 1977; Cevallos-Ferriz & Werber, 1980; Acosta, Garcia Hernandez & Checa, 1988; Shapiro & Awramik, 2000). The transition from LLH morphology in Subfacies 5 to SH morphology in Subfacies 6 suggests an increase in environmental hydrodynamic energy (cf. Acosta, Garcia Hernandez & Checa, 1988), which can be caused by decrease in depth, increasing the influence of the waves.

Other factors that can generate the presence of columnar structures are the desiccation, the heavy sedimentation and the induration. However, these were discarded since there are no evidence of them in the study of the microstructure.

The microstructure in this subfacies presents no change between “build up” and “sheets areas”. The presence of alternating simple lamination (*sensu* Monty, 1976) with abundant, micritic, grain-less laminae suggests that the accretionary process is controlled by in situ, microbially influenced, carbonate precipitation (Reid et al., 2000), and indicates cyclical variations in environmental factors throughout the development of this subfacies (Riding, 2008; Decho, 2000; Riding, 2011). The confluence of laminae across intercolumnar space occurs when spaces were filled with sediment and the algal sheath from the column is able to extend out-ward to connect with the sheath on adjacent columns (Logan, Rezak & Ginsburg, 1964).

On the other hand, the interruption in the vertical development of the lamination can be defined as hiatuses, which reflect variations in environmental controls (Suarez-Gonzalez et al., 2014). Although the hiatus may be due to the action of different factors, the presence of calcareous sandstones in them suggests moments of great influence by the waves and possible temporary subaerial exposure.

Based on this, it can be interpreted that the Subfacies 6 was formed in a shallow, low microtidal (<2 m tidal range) environment, the increase in the hydrodynamic energy of the environment could be explained by a decrease in depth, which increased the influence of the waves on the system.

In Subfacies 7, the mesostructure has composed of laminated columns of the type SH-V (Logan, Rezak & Ginsburg, 1964). The passage of an internal structure of type SH-C (Subfacies 6) to one of the SH-V type (Subfacies 7) suggest an increasing in the “attraction distance” that leads to larger protection zones product of an increase in the hydrodynamic energy with respect to the Subfacies 6 (Cevallos-Ferriz & Werber, 1980; Kah & Bartley, 2004; Kah et al., 2006). This increase in the hydrodynamic energy is also evident in the absence of confluence of laminae across the intercolumnar space throughout all the Subfacies 7 (Cevallos-Ferriz & Werber, 1980).

The increase in the hydrodynamic energy can be caused by a decrease in depth, increasing the influence of the waves. This is supported by the closeness of these facies to the e-M discontinuity.

Other factors that can generate the presence of columnar structures like the desiccation, the heavy sedimentation and the induration; were discarded since there is no evidence of them in the study of the microstructure. Which like in the Subfacies 6 presents no change between “build up” and “sheets” areas.

Abundant micritic grain-less laminae in alternating simple lamination (*sensu* Monty, 1976) indicates cyclical variations in environmental factors throughout the development of this subfacies (Riding, 2008; Decho, 2000; Riding, 2011).

As in the Subfacies 6, the interruption in the vertical development of the lamination can be defined as hiatuses, which reflect variations in environmental controls (Suarez-Gonzalez et al., 2014). The presence of calcareous sandstones in them suggests moments of great influence by the waves and possible temporary subaerial exposure.

It can be concluded that the Subfacies 7 grew up in a shallow, low microtidal (<2 m tidal range) environment, similar to that of Subfacies 6 but with a slightly higher energy gradient. The increase in the hydrodynamic energy of the environment could be explained by a decrease in depth (showing a depth even lower than in the Subfacies 6), which increased the influence of the waves on the system.

The end of stromatolitic growth is generally attributed to changes in environmental conditions (for example, as a result of shallowing of the water body), generating a prevalence of physical and chemical factors over biological factors. In this case, the fall in sea level associated with the Messinian Salinity Crisis caused the emersion of the study area, and the generation of a lowstand erosional surface (e-M unconformity) (Soria et al., 2008a, 2008b).

## Conclusions

The CAM sequence, located in the Sierra del Colmenar, presents a well developed stromatolitic level, both laterally and vertically. The predominance of micritic grain-less laminae during the development of the stromatolite suggest microbially influenced carbonate in-situ precipitation as the main growth factor in an environment with relatively low hydrodynamic energy, probably with high salinities.

External morphology of the stromatolites in the CAM sequence is Domical Colloform, suggesting a growth in lower intertidal to subtidal zone at depths of 0.5–2 m.

The macrostructure architecture is influenced by currents and associated wave energy as relevant factors. It develops continuously but describing a doming type morphology (build up and sheets areas) which helps to reduce the physical stress and the erosion, acting as a palaeogeographic barrier near the coastline. Domical shapes were formed as a stromatolite response to minimize the force of the water acting upon them, while the “sheets areas” would act as large discharge areas.

In general, a similar environment is suggested for the formation of the entire stromatolitic level. However, the variation in internal morphology over vertical stromatolite growth provides evidence of minor changes in the physical environment during the development of each stromatolitic subfacies, largely a product of variations in the depth of the environment.

The upper part of Subfacies 1 is where stromatolitic lamination is first visible, marking the beginning of a subaqueous environment with relatively low hydrodynamic energy, which is maintained throughout the development of Subfacies 2.

Subfacies 3 represents a sudden loss of depth (with respect to Subfacies 2) and an increase in hydrodynamic wave energy. However, at the transition to Subfacies 4, a progressive increase in depth begins, in conditions of relatively low hydrodynamic energy (favored or not by restriction of the paleogeographic setting), giving rise to LLH-S lamination cycles. This increase in depth reaches a maximum during Subfacies 5, providing more accommodation space, larger vertical development and a change to LLH-C mesostructure in a subtidal environment.

The transition from Subfacies 5 to 6 indicates a gradual decrease in depth, reaching an intertidal wave-dominated environment, resulting in SH-C type internal structure. During the formation of Subfacies 7, shallowing continued, generating columnar SH-V type structures in response to the increase in wave action at depths of less than two m.

Continuous shallowing puts an end to stromatolite growth and generated deposition/sedimentation of calcareous-sedimentary material on “sheets areas” spaces. Finally, the sequence was subjected to sub-aerial exposure, giving rise to an erosive process, as represented by the e-M discontinuity.

## Additional Information and Declarations

### Data Availability

The following information was supplied regarding data availability: The sampled material is permanently deposited in the paleontological repository of the Department of Earth and Environmental Sciences, University of Alicante and can be consulted by researchers by referencing “Messinian stromatolites-section CAM.”
